# Supplementary material for: Feedback inhibition underlies new computational functions of cerebellar interneurons
Source: eLife. 2022 Dec 8;11:e77603. doi: 10.7554/eLife.77603 (PMC9771357; doi:10.7554/eLife.77603)
Supplement: Transparent reporting form [file elife-77603-transrepform1.docx]

***eLife’s* transparent reporting form**

We encourage authors to provide detailed information *within their submission* to facilitate the interpretation and replication of experiments. Authors can upload supporting documentation to indicate the use of appropriate reporting guidelines for health-related research (see [EQUATOR Network](http://www.equator-network.org/%20)), life science research (see the [BioSharing Information Resource](https://biosharing.org/)), or the [ARRIVE guidelines](http://www.plosbiology.org/article/info:doi/10.1371/journal.pbio.1000412) for reporting work involving animal research. Where applicable, authors should refer to any relevant reporting standards documents in this form.

If you have any questions, please consult our Journal Policies and/or contact us: [editorial@elifesciences.org](mailto:editorial@elifesciences.org).

**Sample-size estimation**

- You should state whether an appropriate sample size was computed when the study was being designed
- You should state the statistical method of sample size computation and any required assumptions
- If no explicit power analysis was used, you should describe how you decided what sample (replicate) size (number) to use

Please outline where this information can be found within the submission (e.g., sections or figure legends), or explain why this information doesn’t apply to your submission:

We did not compute sample size in advance because we had no a priori knowledge of effect size. Our goal was to investigate the organization and properties of a specific circuit between PCs and PC-MLIs and determine its roles within the cerebellum, rather than detecting effects between control and experimental groups. Our sample sizes are considered adequate for the experiments and are similar to those reported in previous publications (Kim et al., 2014; Halverson et al., 2015). In every case, N numbers and statistical tests are provided in the Results section and/or figure legends.

**Replicates**

- You should report how often each experiment was performed
- You should include a definition of biological versus technical replication
- The data obtained should be provided and sufficient information should be provided to indicate the number of independent biological and/or technical replicates
- If you encountered any outliers, you should describe how these were handled
- Criteria for exclusion/inclusion of data should be clearly stated
- High-throughput sequence data should be uploaded before submission, with a private link for reviewers provided (these are available from both GEO and ArrayExpress)

Please outline where this information can be found within the submission (e.g., sections or figure legends), or explain why this information doesn’t apply to your submission:

Each experiment was biologically replicated more than 4 times.

Biological replicates are electrophysiologically recorded MLIs. Usually a few PC-MLIs were detected per animal in vitro patch clamp recordings due to the relative sparseness of cells compared with other MLIs. Information regarding the number of biological replicates are included in the text and/or figure legend. The electrophysiological data were analyzed if the series resistance of MLIs were not significantly changed (>20%). No analyzed data were excluded.

In the case of tetrode recordings in vivo, any recording that lost during a recording session was not included in the analysis.

**Statistical reporting**

- Statistical analysis methods should be described and justified
- Raw data should be presented in figures whenever informative to do so (typically when N per group is less than 10)
- For each experiment, you should identify the statistical tests used, exact values of N, definitions of center, methods of multiple test correction, and dispersion and precision measures (e.g., mean, median, SD, SEM, confidence intervals; and, for the major substantive results, a measure of effect size (e.g., Pearson's r, Cohen's d)
- Report exact p-values wherever possible alongside the summary statistics and 95% confidence intervals. These should be reported for all key questions and not only when the p-value is less than 0.05.

Please outline where this information can be found within the submission (e.g., sections or figure legends), or explain why this information doesn’t apply to your submission:

General information on data analysis was given in the Methods section. Every section and/or figure legend has statistical information (if any) concerning the data representation, and details of tests.

(For large datasets, or papers with a very large number of statistical tests, you may upload a single table file with tests, Ns, etc., with reference to sections in the manuscript.)

**Group allocation**

- Indicate how samples were allocated into experimental groups (in the case of clinical studies, please specify allocation to treatment method); if randomization was used, please also state if restricted randomization was applied
- Indicate if masking was used during group allocation, data collection and/or data analysis

Please outline where this information can be found within the submission (e.g., sections or figure legends), or explain why this information doesn’t apply to your submission:

No group allocation was needed.

**Additional data files (“source data”)**

- We encourage you to upload relevant additional data files, such as numerical data that are represented as a graph in a figure, or as a summary table
- Where provided, these should be in the most useful format, and they can be uploaded as “Source data” files linked to a main figure or table
- Include model definition files including the full list of parameters used
- Include code used for data analysis (e.g., R, MatLab)
- Avoid stating that data files are “available upon request”

Please indicate the figures or tables for which source data files have been provided:

Source data files are provided for Figure 1, 2, 3, 4, 7, 9 and 10.

Source code is available at [https://github.com/mauk-lab-utexas/CBMSim](https://nam12.safelinks.protection.outlook.com/?url=https%3A%2F%2Fgithub.com%2Fmauk-lab-utexas%2FCBMSim&data=05%7C01%7C%7Cdf613acc2cd44269f4a708daa553da46%7C31d7e2a5bdd8414e9e97bea998ebdfe1%7C0%7C0%7C638004076804610202%7CUnknown%7CTWFpbGZsb3d8eyJWIjoiMC4wLjAwMDAiLCJQIjoiV2luMzIiLCJBTiI6Ik1haWwiLCJXVCI6Mn0%3D%7C3000%7C%7C%7C&sdata=1Xq2JT5Br5chspo8OJQw1N5qFT9P6LJ4qO%2Bw2WSo8VE%3D&reserved=0)
